# Os Benefícios dos Exercícios Físicos no Câncer de Mama

**DOI:** 10.36660/abc.20220086

**Published:** 2022-11-23

**Authors:** Milena dos Santos Barros Campos, Roberta Helena Fernandes Feitosa, Carolina Christianini Mizzaci, Maria do Rosário Toscano von Flach, Betty Janny Maia Siqueira, Luiz EduardoMastrocola

**Affiliations:** 1 Clínica e Hospital São Lucas RedeD'Or São Luiz Aracaju SE Brasil Clínica e Hospital São Lucas, RedeD'Or São Luiz, Aracaju, SE – Brasil; 2 Divisão de Cardiologia do Hospital Universitário de Sergipe Aracaju SE Brasil Divisão de Cardiologia do Hospital Universitário de Sergipe, Aracaju, SE – Brasil; 3 Secretaria Municipal de Saúde Gerência dos Sistemas de Informações Epidemiológicas Goiânia GO Brasil Gerência dos Sistemas de Informações Epidemiológicas - Secretaria Municipal de Saúde, Goiânia, GO – Brasil; 4 Instituto Dante Pazzanese de Cardiologia São Paulo SP Brasil Instituto Dante Pazzanese de Cardiologia, São Paulo, SP – Brasil; 5 Fundação Bahiana para Desenvolvimentodas Ciências Salvador BA Brasil Fundação Bahiana para Desenvolvimentodas Ciências (FBDC), Salvador, BA – Brasil; 6 Universidade de Pernambuco Recife PE Brasil Universidade de Pernambuco, Recife, PE – Brasil

**Keywords:** Neoplasias da mama, Técnicas de Exercício e Movimento, Exercício, Dieta Saudável

## Abstract

O câncer de mama é a neoplasia mais prevalente nas mulheres, em torno de 30% de todos os tipos de câncer, enquanto no sexo masculino é responsável por apenas 1% dos casos. No mundo, é a primeira causa de morte por câncer em mulheres. A incidência e a mortalidade variam de acordo com a etnia, região territorial e nível socioeconômico. Em virtude da baixa prevalência do câncer de mama em homens e a escassez de literatura, as condutas são baseadas em extrapolações dos estudos no sexo feminino. As evidências científicas sugerem efeitos benéficos dos exercícios físicos na prevenção, durante o tratamento e no pós-tratamento do câncer de mama. Além do combate ao sedentarismo, é importante manter um peso saudável, limitar o consumo de álcool, e seguir dieta balanceada, rica em frutas, vegetais, grãos e fibras e reduzida em carnes vermelhas. As ações dos exercícios não se restringem ao câncer de mama, mas têm impacto importante no controle dos fatores de risco modificáveis, diminuindo a incidência das doenças cardiovasculares e a mortalidade por causas globais e cardíaca.

## Introdução

O câncer de mama é a neoplasia mais incidente em mulheres no mundo, com aproximadamente 2,3 milhões de casos novos em 2020, o que representa 24,5% dos tipos de câncer.^1^ No Brasil, excluindo os tumores de pele não melanoma, o câncer de mama é o mais frequente em mulheres, com taxas mais altas no Sul e Sudeste.^2^ As justificativas potencialmente atribuídas para a maior ocorrência nessas regiões são o maior índice de desenvolvimento humano e expectativa de vida, prevalência elevada da raça branca, o estilo de vida, gestação mais tardia e menor número de filhos.^3,4^ Para o ano de 2022, foram estimados 66 280 casos novos no Brasil.^2^

Quanto à mortalidade, a neoplasia mamária é a primeira causa de morte por câncer na população feminina no Brasil, exceto na região Norte, onde o câncer do colo do útero ocupa essa posição. As maiores taxas de mortalidade são nas regiões Sudeste e Sul, que são crescentes a partir dos 40 anos de idade.^5^ Uma possibilidade a ser questionada em localidades com menores taxas de mortalidade, como o Norte, é o subdiagnóstico do câncer de mama.

Causas genéticas, como as mutações dos genes BRCA1 e BRCA2, são responsáveis por 5 a 10% de todos os casos de câncer de mama e ovário, com maior contribuição dos fatores ambientais e do estilo de vida na patogênese destes tumores.^6^ BRCA1 e BRCA2 produzem proteínas supressoras do tumor. Essas proteínas reparam o DNA danificado e, portanto, desempenham um papel na garantia da estabilidade do material genético de cada célula. Quando um desses genes sofre uma mutação ou alteração, de forma que seu produto proteico não funcione corretamente, o dano ao DNA pode não ser reparado de maneira adequada. Como resultado, as células têm maior probabilidade de desenvolver alterações genéticas que podem levar ao desenvolvimento do câncer.^7^

A adoção de estilo de vida saudável é importante na prevenção do câncer de mama, abrangendo nutrição adequada (maior consumo de frutas, vegetais e grãos integrais e menor consumo de carne vermelha), controle do peso, redução de ingesta alcóolica e prática de exercícios físicos (EF).^8^ Seus efeitos parecem não se restringir à prevenção, mas também ao controle da doença, pois estudos experimentais têm demonstrado ações na cinética de formação, crescimento e recorrência do tumor.^9^

O terceiro Consenso do *World Cancer Research Fund*^10^ e o Guia de Atividade Física e Câncer da Sociedade Brasileira de Oncologia^11^ abordam a importância da mulher ser fisicamente ativa na prevenção e durante o tratamento do câncer de mama, trazendo diversos tipos de atividade física, desde atividades domésticas (como a jardinagem), ocupacionais e recreacionais, até as sistematizadas, apropriadamente denominadas como EF, que são atividades com prescrição estabelecida de frequência, intensidade, tempo e tipo (aeróbico, resistido e combinado).

### Mecanismos de ação dos exercícios físicos na progressão tumoral

Os EF promovem diferentes mecanismos orgânicos e biológicos que podem participar no controle do desenvolvimento de tumores variados. Tais respostas são provenientes de alterações metabólicas e dos hormônios sexuais, além da modulação da inflamação sistêmica.^12^ No entanto, o potencial de atingir diretamente a progressão tumoral foi mais recentemente relacionado às alterações na vascularização e fluxo sanguíneo dos tumores,^13^ à utilização de substratos pelas células neoplásicas, às relações proteicas entre o câncer e o tecido muscular, e à regulação da função imunológica mediados pelos EF.^14–16^

O microambiente tumoral trabalha para cooptação e desvio da ação de células imunoinflamatórias e estromais a seu favor.^17^ Enquanto a ação aguda e transitória, principalmente de linfócitos e macrófagos, é fator de controle e reparo do dano tecidual, a inflamação crônica e infiltração macrofágica no tecido promovem a progressão tumoral.^18^

**Figura 1:**
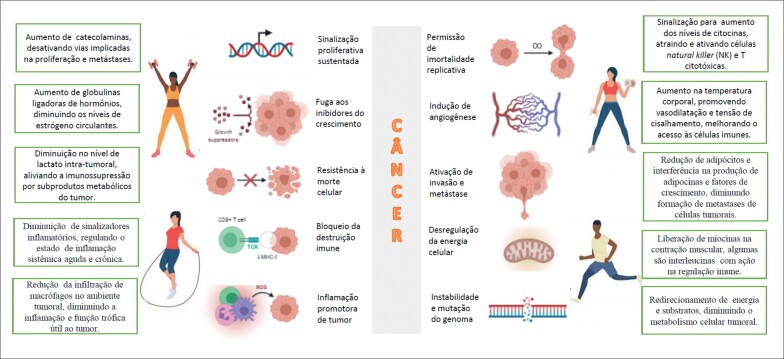
Esquematização dos mecanismos pelos quais o exercício físico pode exercer influência sobre os hallmarks do câncer no câncer de mama. Adaptado de “Hallmarks of cancer”, by BioRender.com

O desenvolvimento de terapias contra o câncer é baseado nos *hallmarks* (conjunto de capacidades adquiridas pelas células humanas no processo de transformação neoplásica) propostos por Weinberg a partir do ano 2000.^19^ Também é a partir desses conceitos que vem sendo estudadas as possíveis ações e adaptações pelas quais o EF pode influenciar nessas marcas do desenvolvimento tumoral (Figura 1).

Os resultados dos estudos pré-clínicos indicam que esses efeitos moleculares resultantes de cada sessão de EF se sobrepõem aos controles dos fatores hormonais e insulínicos.^20^ Durante a execução dos EF, esses fatores exercem ação imediata no metabolismo tumoral, e o treinamento de longo prazo leva a adaptações metabólicas e imunogênicas que contribuem para lentificar a progressão do tumor.^21^

Evidências de que o EF diminui a progressão do tumor maligno surgiram em modelos animais.^12,22,23^ Recentemente, estudos clínicos vêm identificando ações celulares e moleculares semelhantes do exercício em pacientes com câncer, incluindo o de mama,^24,25^ porém não há ainda estudos que esclareçam a relevância clínica desses resultados.

### Exercícios físicos na prevenção do câncer de mama

Um dos primeiros e maiores estudos prospectivos que abordaram a atividade física e o câncer de mama foi o “*Nurses Health Study*”, publicado em 1999. O estudo avaliou 121701 enfermeiras de 30 a 55 anos, no seguimento de 16 anos, e demonstrou que as praticantes de atividade física moderada ou vigorosa por sete ou mais horas por semana evidenciavam redução de risco próximo a 20% para o desenvolvimento de câncer de mama, em comparação com as que mantinham essa atividade física por menos de uma hora na semana [risco relativo de 0,82 - intervalo de confiança (IC) de 95%, 0,70-0,97]. O benefício foi observado em mulheres no período pré e no pós-menopausa.^26^

A literatura sugere que mulheres praticantes de EF regulares apresentam menor risco de câncer de mama, entre 10 e 25%, quando comparadas às mulheres menos ativas.^27,28^ As associações parecem ser mais fortes em atividades mantidas regularmente ao longo da vida e no período pós-menopausa, em mulheres com peso normal, não brancas, sem histórico de câncer de mama na família e que tiveram filhos.^29^ Em pacientes portadores de alteração dos genes BRCA1 e/ou BRCA2, as evidências do impacto dos EF na redução do câncer de mama ainda são limitadas, havendo a necessidade de estudos maiores.^30^

Estudo de metanálise publicado em 2013, com inclusão de 31 trabalhos prospectivos, verificou associação significativa entre atividade física e redução de câncer de mama, com risco relativo combinado de 0,88 (0,85-0,91), dentro de IC de 95%. Análise de dose resposta sugeriu que o risco de câncer de mama reduziu 2% a cada aumento de 25 equivalentes metabólicos (MET)-horas/semana em atividades físicas não ocupacionais (aproximadamente 10 horas por semana de atividades domésticas), 3% para cada 10 MET-horas/semana em atividades recreacionais (equivalente à caminhada de 3 quilômetros por hora, durante 4 horas/semana) e 5% para cada 2 horas/semana em atividades recreacionais moderadas e intensas.^31^ Outros trabalhos reafirmaram tais resultados, sugerindo maior redução do risco de câncer de mama com níveis mais intensos de EF.^28,32^ A dose exata e o tipo de atividade necessária para reduzir o risco de câncer de mama ainda não foram totalmente esclarecidos.

Em uma revisão abrangente,^33^ o estudo tipo *umbrela* sobre atividade física na incidência e mortalidade por câncer, os resultados foram sugestivos para redução do risco de câncer de mama na população geral. No entanto, a classificação da atividade física entre os estudos foi heterogênea e a maioria das revisões foi baseada em estudos observacionais, principalmente os tipos coorte, onde é difícil o controle do viés de seleção, porque comportamentos de estilo de vida saudável tendem a se agrupar. Por exemplo, uma pessoa que adota hábitos saudáveis, pratica atividade física, alimenta-se adequadamente, tem peso mais perto do ideal e não fuma. Para minimizar as limitações deste tipo de estudo, as diretrizes vêm se apoiando principalmente em estudos de coorte com maior amostra da população.^33^

O papel dos EF na prevenção de câncer de mama parece estar atrelado à redução da atividade estrogênica, da resistência à insulina, da inflamação e do estresse oxidativo.^34^ O estrogênio está relacionado à indução de proliferação celular e desenvolvimento tumoral. Os EF aumentam a proteína ligadora dos hormônios sexuais, reduzindo os níveis circulantes de estrogênio conforme descrito na Figura 1. EF contribuem na redução da massa gorda, principalmente na diminuição da gordura visceral, e melhora da sensibilidade celular à insulina e consequente redução do seu nível sérico. A insulina está envolvida na ativação da aromatase e aumento do estrogênio, além de apresentar ação mitogênica. Os EF têm efeitos imunomodulatórios, com aumento da imunidade inata e adquirida e melhora dos mecanismos de reparo do DNA, diminuindo a carcinogênese.^35^ Mais pesquisas são necessárias para entender completamente os mecanismos pelos quais a atividade física pode reduzir o risco de câncer de mama.

### Recomendações gerais para a prática de atividades físicas na prevenção e controle do câncer de mama

Em 2020, a Organização Mundial da Saúde (OMS) e o Ministério da Saúde do Brasil recomendaram, tanto para a população adulta (18 a 64 anos) em geral, quanto para sobreviventes de câncer de mama, a prática de pelo menos 150 a 300 minutos semanais de atividade física de intensidade moderada, ou pelo menos 75 a 150 minutos semanais de intensidade vigorosa, ou, ainda, uma combinação equivalente de atividades moderadas e vigorosas no decorrer da semana.^11,36,37^

A Tabela 1 resume, com base no Guia de Atividade Física para a População Brasileira, a descrição das diferentes intensidades das atividades físicas.

**Tabela 1:** Intensidades das atividades físicas recomendadas para prevenção e controle do câncer de mama

Intensidade	Descrição	Exemplos
Leve <3METs (Equivalentes metabólicos)	Exige mínimo esforço físico, com pequeno aumento da FR e da FC. Numa escala de 0 a 10, a percepção de esforço é de 1 a 4. É possível respirar tranquilamente e conversar normalmente durante a movimentação ou, até mesmo, cantar	Manter-se de pé ou sentado, lavar pratos, realizar trabalhos manuais
Moderada 3 a 5,9 METs	Exige mais esforço físico, com incrementos perceptíveis, porém moderados, da FR e da FC. Numa escala de 0 a 10, a percepção de esforço é 5 a 6. É possível conversar com dificuldade durante a movimentação, mas não cantar	Andar a mais de 5 km/h, andar de bicicleta a menos de 16 km/h, jogar tênis em duplas e dança de salão.
Vigorosa > 6 METs	Exige um grande esforço físico, com incrementos intensos da FR e da FC. Numa escala de 0 a 10, a percepção de esforço é 7 a 8. Não é possível conversar durante a movimentação.	Correr, andar em terreno com inclinação, andar de bicicleta a mais de 16 km/h, dança aeróbica

Fonte: Adaptado do Guia de Atividade Física para a População Brasileira, Ministério da Saúde, BRASIL.^37^ FR: frequência respiratória; FC: frequência cardíaca.

É importante a diferenciação entre a prática de atividades físicas (movimentos voluntários do corpo, com gasto de energia acima do nível de repouso) e de exercícios físicos (atividades físicas planejadas, estruturadas e repetitivas que têm como objetivos fundamentais a melhoria da aptidão cardiorrespiratória, força, flexibilidade e equilíbrio). É recomendável que os EF sejam supervisionados por profissional de educação física ou fisioterapeuta e que todos os programas contenham atividades com componentes aeróbicos (caminhar, andar de bicicleta, dançar, correr, nadar), de força muscular (musculação, Pilates, exercícios funcionais) e de amplitude articular (alongamentos, ioga, *tai-chi*).^11^

Exercícios aeróbicos aumentam os níveis de beta-endorfinas periféricas, correlacionadas à queda da atividade simpática sistêmica e melhora da atividade serotoninérgica refletidas na atividade das junções neuromusculares. Exercícios de resistência produzem melhor sincronização, recrutamento e excitabilidade das unidades de placas motoras. Por fim, os exercícios de flexibilidade podem proporcionar melhor controle sobre as estruturas articulares e partes moles.^38,39^

A seguir, detalhamos particularidades da prática de atividades e EF durante o tratamento e o acompanhamento de sobreviventes pós-tratamento para câncer de mama.

### Exercícios físicos durante o tratamento do câncer de mama

O tratamento do câncer de mama deve ser individualizado segundo condições da paciente como idade, *status* hormonal, comorbidades, estilo de vida e escolhas pessoais, bem como norteado por dois parâmetros fundamentais que delineiam o prognóstico: a extensão da doença (estadiamento) e o tipo do tumor. Em linhas gerais, pode ser dividido em tratamento local, que envolve cirurgias e radioterapia (além de reconstrução mamária) e tratamento sistêmico com quimioterapia, hormonioterapia e terapia biológica.^40^

A quimioterapia se associa à fadiga, anorexia, anemia, neutropenia, trombocitopenia, neuropatias periféricas e em alguns casos, cardiotoxicidade.^41,42^ Os efeitos colaterais da hormonioterapia incluem ganho de peso, artralgia, mialgia, perda óssea, efeitos no sistema cardiovascular e alterações no perfil lipídico.^43^ Entre as sequelas da irradiação, estão danos cardíacos e pulmonares, linfedema, plexopatia braquial, além de doenças malignas secundárias.^44^ Associadas às repercussões físicas, pode haver alterações emocionais como depressão, ansiedade, baixa autoestima, além de percepção negativa da imagem corporal, uma vez que o câncer de mama acomete importante símbolo da feminilidade, sensualidade, sexualidade e também da maternidade.^45^

Seis meses após o diagnóstico aproximadamente 90% das mulheres manifestam ao menos um dos sintomas adversos da terapêutica antineoplásica; 60% cursam com múltiplos efeitos, que influenciam não só o tratamento e a qualidade de vida dessas pacientes, como também taxas de sobrevida. Há relatos que após seis anos do fim do tratamento, até 30% das mulheres persistem com queixas relacionadas às diferentes terapias empregadas.^46^

A atividade física é segura e pode ser realizada durante os diferentes processos de tratamento, resultando em melhora da qualidade de vida, funcionalidade global e redução de sintomas psicológicos relacionados à doença e seus tratamentos.^47,48^

**Figura 2:**
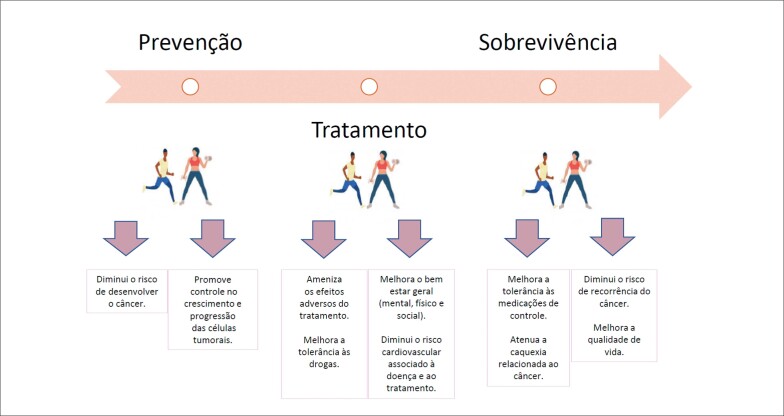
Resultados clínicos do exercício na linha do tempo do câncer de mama

A dor é um dos sintomas mais comuns nas pacientes com câncer de mama, 30-60% apresentam o sintoma com intensidade moderada a acentuada, resultando em limitação ou interrupção da atividade física durante e após as intervenções terapêuticas.^39,49^ As manifestações álgicas, tendem a decair com o treinamento físico, com implicações diretas no ganho de força, capacidade cardiorrespiratória e flexibilidade e ainda, na redução das taxas de fadiga, tempo de permanência hospitalar, ansiedade, depressão, distúrbios do sono, náuseas e vômitos.^50,51^ A Figura 2 resume os efeitos clínicos finais dos EF durante o tratamento, assim como nas demais fases do câncer de mama.

Van Waart et al.,^52^ demonstraram que o treinamento aeróbico em pacientes submetidos à quimioterapia associou-se à melhora da funcionalidade física, mantendo a aptidão cardiorrespiratória, facilitando a reincorporação às atividades laborativas durante e após o tratamento, e ainda, reduzindo a incidência de náuseas, vômitos e dor, quando comparado ao grupo sem protocolos de treinamento físico.^52^

Adicionalmente, um estudo multicêntrico^53^ avaliou o efeito da atividade física em 301 pacientes durante tratamento quimioterápico para câncer de mama, evidenciando-se melhora da aptidão física em todos os tipos de treinamento aplicados: grupo padrão (três sessões de 25-30 min/semana), grupo alto volume (três sessões de 50-60 min/semana) e treinamento combinado (aeróbico associado ao resistido). O grupo do exercício de alto volume foi mais efetivo em melhora da qualidade de vida, da capacidade aeróbica e no controle da dor e sintomas endócrinos (fogachos por exemplo). No entanto, o grupo de treinamento combinado apresentou melhor evolução quanto ao ganho de força muscular.^53^

Em pacientes na vigência de quimioterapia, os programas de exercícios resistidos estão mais associados à melhora da autoestima, força muscular e composição corporal, sem desencadear ou agravar linfedemas ou outros efeitos adversos em pacientes submetidas à cirurgia.^54,55^ Recomenda-se a avaliação da mobilidade do braço antes do início de EF para os membros superiores. Além disso, é importante a avaliação específica para neuropatias periféricas, doenças musculoesqueléticas e risco de fraturas, principalmente nas pacientes em uso de terapia hormonal ou com doença óssea metastática.^46,56,57^

A combinação dos três tipos de propostas (exercícios aeróbicos, resistidos e flexibilidade) tem contribuído em maior escala para o controle da dor e fadiga. A melhora da função cardiorrespiratória, pelo aumento da capacidade aeróbica (consumo de oxigênio máximo) nos treinos combinados, pode ser explicada pela significante interação ventilação-perfusão e capacidade oxidativa musculoesquelética.^55^ Isso pode ter importante papel no manejo da desordem estrutural relacionada à toxicidade secundária ao tratamento quimio e radioterápico.^46^

A quimioterapia também pode provocar efeitos cardiotóxicos diretos e indiretos, ocorrendo aceleração do envelhecimento geral e vascular, com consequente declínio da reserva cardiopulmonar. Tanto a própria doença como o tratamento podem contribuir para o ganho de peso e para a redução da atividade física,^58^ aumentando potencialmente o risco de doenças cardiovasculares (DCV). Estudos de prevenção secundária ratificaram a melhora da função cardiopulmonar com a aplicação de programas de treinamento físico em mulheres com câncer de mama.^59^

A cardiotoxicidade, associada a fatores psicoemocionais, interfere sobre o equilíbrio autonômico e consequentemente na mortalidade por DCV.^60^ Demonstrou-se que, em pacientes tratadas em estágios iniciais da doença, ocorre incremento sustentado da função simpática e redução da ação parassimpática no nó sinoatrial.^61^ Ressaltam-se outros estudos que evidenciaram a redução da variabilidade da frequência cardíaca e da sensibilidade barorreflexa entre mulheres com histórico de câncer de mama.^61–63^

Além da regulação autonômica, outro fator importante para o desenvolvimento de DCV é a disfunção endotelial. Metanálise recente^64^ avaliou 163 pacientes de quatro estudos (dois para câncer de mama, dois para câncer de próstata). O exercício aeróbico melhorou a função vascular e o consumo de oxigênio pico. Os dados acima apresentados destacam a importância do EF como coadjuvante do tratamento para câncer de mama, sobretudo no que se refere à abordagem de seus efeitos colaterais.

No que tange à mortalidade geral e específica, a evidência acumulada até o momento parece indicar efeito favorável da atividade física moderada a vigorosa, e evidências preliminares sugerem redução no risco de recorrência e progressão do câncer de mama associada a essas práticas.^11^ Recente publicação avaliou revisões sistemáticas sobre atividade física e redução da mortalidade tanto por todas as causas quanto especificamente por câncer de mama, considerando relação dose-resposta (inclusive independentemente do índice de massa corporal); a “certeza da evidência” foi classificada como moderada. No que se refere ao domínio ou tipo da atividade física e mortalidade, a certeza da evidência foi classificada como baixa, não sendo possível, até então, identificar modalidades que possam ter maior impacto sobre esse desfecho. A mesma publicação também avaliou o balanço entre riscos e benefícios, valores e preferências de pacientes, recursos necessários para se realizar o que se recomenda, equidade, aceitabilidade e viabilidade da recomendação, classifica como “forte” a “força de recomendação” de EF para aumentar a sobrevida de câncer de mama.^11^ Estudos futuros poderão modificar a qualidade das evidências disponíveis até então; é um campo aberto ainda para pesquisas.

### Exercícios físicos no pós-tratamento do câncer de mama

Para as sobreviventes do câncer de mama, EF têm sido amplamente recomendados, sendo vinculado não só com melhoria na qualidade de vida, mas também com possível aumento da sobrevida.^65–67^ Em estudo prospectivo que incluiu 2987 mulheres com câncer de mama estágios I, II ou III entre 1984 e 1998 e que foram acompanhadas até o óbito ou junho de 2002, demonstrou que a atividade física após o diagnóstico de câncer de mama pode reduzir o risco de morte por esta doença. O maior benefício ocorreu em mulheres que realizaram o equivalente a caminhada de três a cinco horas por semana em ritmo médio.^65^

Após o término do tratamento, o objetivo é reabilitar a paciente para que possa retornar o mais rápido possível às suas atividades habituais. A prática regular de EF pode contribuir para o bem estar físico, psicológico e para melhor qualidade de vida, sendo uma das principais recomendações para evitar o aparecimento de condições crônico degenerativas, e não seria diferente para as pacientes que acabaram de enfrentar tratamento oncológico.^68^ Evidências mostram que os EF proporcionam impacto positivo na sobrevida e minimizam a morbidade relacionada ao câncer de mama.^65^ Apesar de dados favoráveis, a prática é limitada devido a barreiras como fadiga, ausência de motivação, perda da autoconfiança, acompanhamento inadequado, falta de suporte familiar e falta de orientações.

Incentivar as mulheres no período após o tratamento, a adotarem estilo de vida saudável, evitando o consumo excessivo de álcool, aumentando a ingestão de frutas e vegetais, além de maior volume de atividade física, pode ser importante para melhorar sua saúde e a qualidade de vida.^69^ Níveis mais elevados de EF representam comportamento de saúde modificável que pode aliviar sequelas da doença e ajudar as mulheres a retornarem ao estado de saúde que tinham antes de receber o diagnóstico e o tratamento.^70^ Desta forma, as recomendações atuais relacionadas aos EF para as sobreviventes voltam-se ao retorno às atividades diárias normais o mais rápido possível após a cirurgia, à manutenção do gasto metabólico durante e após as terapêuticas implementadas, além da clássica orientação para a atividade aeróbica semanal.^57^

Adicionalmente demonstrou-se que a falta de atividade física está relacionada ao ganho de peso após o diagnóstico o que, por sua vez, tem sido associado à menor sobrevida em alguns estudos.^71,72^ Mulheres mais ativas possuem menor propensão a ganhar peso após o diagnóstico, melhorando assim suas chances de sobrevivência.^65,73^

A obesidade está relacionada a taxas aumentadas de mortalidade devido ao câncer de mama (13-20%) e mortalidade por todas as causas (14-70%).^74–77^ A obesidade também foi associada a risco duas vezes maior de câncer de mama contralateral na pós-menopausa e à maior ocorrência, próxima a 60%, de outros cânceres.^76^ Portanto, o índice de massa corporal, dentro da normalidade, pode reduzir o risco do aparecimento de nova neoplasia mamária na pós-menopausa, de outros tipos de câncer e mortalidade por todas as causas.^75,76,78^

Giallauria et al.,^61^ avaliaram se EF poderiam melhorar a função autonômica de mulheres com história de câncer de mama primário invasivo. Foram incluídas 51 pacientes inscritas no estudo clínico denominado “DIANA”, divididas em dois grupos. Grupo 1 (n=25) seguiu programa formal de EF com intensidade moderada – carga de trabalho aplicada em 70 ± 2% do consumo de oxigênio (VO_2_ pico inicial) e frequência de três sessões por semana, em bicicleta ou esteira, com duração de 12 semanas, seguido por uma sessão por semana até um ano de acompanhamento. O Grupo 2 ou controle (n=26) não participou em programas formais de exercícios físicos. No início do estudo e após um ano, todas as pacientes foram submetidas a teste cardiopulmonar de exercício. A frequência cardíaca de recuperação (FCR) foi calculada como a diferença entre a frequência cardíaca no pico do exercício e a frequência cardíaca no primeiro minuto pós-esforço. Em comparação ao grupo controle, o grupo 1 apresentou melhora significativa no VO_2_ pico (de 12,6 ± 3,0 para 14,5 ± 3,3 ml.kg^-1^.min^-1^, p<0,001; p<0,001 entre os grupos) e na FCR (de 17,6 ± 6,4 a 23,0 ± 8,3 batimentos/min, p<0,001; p<0,001 entre os grupos). Os autores concluíram que o treinamento físico de intensidade moderada em sobreviventes de câncer de mama pode estar associado à melhora da função autonômica.^62^

As evidências sugerem que os EF podem promover também benefícios fisiológicos e psicológicos positivos entre os sobreviventes do câncer.^70,79^ Em metanálise de estudos randomizados controlados, estruturada por Fong et al.,^70^ observou-se que a prática de EF foi associada a efeitos positivos importantes na função física, peso corporal e qualidade de vida de pacientes que completaram o tratamento para câncer de mama.^70^ Adicionalmente, os resultados relatados em outra revisão sistemática^66^ indicam que os EF podem ter efeitos benéficos na qualidade de vida geral e em certos domínios, como imagem corporal, autoestima, bem-estar emocional, sexualidade, distúrbios do sono, funcionamento social, ansiedade, fadiga e dor.^66^ Ainda, em outra recente revisão sistemática e metanálise^67^ Cochrane, com 63 estudos e 5761 mulheres incluídas, avaliaram-se os efeitos dos EF após tratamento de câncer de mama em comparação a um grupo controle. Novamente demonstrou-se que o grupo que realizou EF, quando comparado ao controle, apresentou melhora da qualidade de vida, da saúde emocional, da ansiedade, da capacidade física, da força muscular e da sensação de cansaço. Além disso, relativamente poucos eventos adversos foram relatados nos diversos trabalhos, sugerindo a prática de EF é segura nesta população.^67^

Para escolhas seguras nas aplicações dos EF, ressalta-se a necessidade do conhecimento pela equipe multidisciplinar de apoio (profissionais de educação física, fisioterapeutas, entre outros) sobre as peculiaridades, implicações e consequências do tratamento do câncer.^57^ As prescrições de EF devem ser de acordo com a capacidade física pré-tratamento e comorbidades do sobrevivente do câncer, resposta à terapêutica e os efeitos negativos imediatos ou persistentes do tratamento.^57^ Recomenda-se especial atenção à neuropatias periféricas e morbidades musculoesqueléticas secundárias, independentemente do tempo desde o tratamento. Se houver terapia hormonal, é recomendada a avaliação do risco de fraturas. Também é indicada a análise específica da mobilidade do braço / ombro antes de exercícios para membros superiores. Deve-se ainda, respeitar o tempo adequado para cicatrização após a cirurgia, que, em caso de mastectomia pode chegar a oito semanas ou até mais.^57^

Os indivíduos com doença metastática óssea exigirão orientação individualizada com o objetivo de determinar limites de segurança antes do início dos exercícios. Para reabilitação desses pacientes, são necessárias modificações no programa estabelecido, com reduções do impacto, da intensidade e do volume, devido ao risco aumentado de fragilidade óssea e fraturas.^57^ Indivíduos com DCV conhecidas (secundárias ao tratamento do câncer ou não) também requerem avaliação médica individualizada inicial relativa à segurança do programa estabelecido de exercícios, bem como maior supervisão e reavaliações em intervalos menores. Devem ser seguidas as orientações de diretrizes para a prescrição de exercícios e reabilitação, em especial considerando-se as contraindicações cardiovasculares e pulmonares impeditivas.^57^

Os sobreviventes de câncer devem evitar a inatividade. Entretanto, a prescrição de exercício baseado na frequência, intensidade, tipo e duração tem se baseado em dados limitados da literatura. A Tabela 2 resume a prescrição de EF recomendada para o pós-tratamento de câncer de mama.^47,57^

**Tabela 2:** Recomendação de prescrição de exercícios físicos no pós-tratamento do câncer de mama

	Frequência	Intensidade	Duração/execução	Qualidade
Aeróbico	Inicialmente, podem acontecer 2 vezes na semana, devendo ser aumentados gradualmente até 3 a 5 vezes na semana.	Orientar sobre a percepção subjetiva do esforço para monitorar a intensidade. Se o EF for tolerado sem sintomas ou efeitos colaterais, a intensidade não precisa ser diferente da população saudável. O exercício aeróbico deve ter intensidade moderada a vigorosa.	O tempo deve ser aumentado de acordo com a tolerância do paciente. Deve-se tentar alcançar duração de 75min/semana de intensidade vigorosa ou 150min/semana de intensidade moderada.	Constituído por atividades rítmicas e prolongadas que trabalhem grandes grupos musculares. Exemplo: natação, caminhada, ciclismo, dança.
Resistido	Realizados 2 a 3 dias na semana	Intensidade moderada (60-70% repetição máxima)	Série de 8 a 12 repetições	Deve incluir cargas, aparelhos contra resistência ou tarefas funcionais com sustentação de carga, trabalhando todos os principais grupos musculares
Flexibilidade	Podem ocorrer diariamente, de acordo com a avaliação da paciente			Deve conter alongamento. Atenção às áreas com restrição de mobilidade devido ao tratamento

Fonte: Adaptado das Diretrizes do American College of Sports Medicine para os testes de esforço e sua prescrição.^*47*^

A progressão do EF poderá ser mais lenta entre os sobreviventes de câncer quando comparados à população saudável, em especial se o exercício prescrito resultar em maior fadiga e sintomas adversos não esperados, que servem como sinais de alerta a limiares da tolerância individual. Não há limite máximo de peso nos exercícios resistidos para o qual os sobreviventes possam progredir. Atenção a manifestações como sintomas em braço e ombro, incluindo linfedema, resultando em redução da resistência ou interrupção de exercícios específicos de acordo com o sintoma relatado.^57^

Apesar dos benefícios derivados da prática regular das atividades mencionadas, não há consenso ou padronização clara sobre a magnitude do benefício, o modo de administração, ou os EF mais eficazes para esta população de pacientes. Mais pesquisas são necessárias para estabelecer a prescrição ideal. Os estudos realizados usaram diferentes modos, frequências, intensidades e durações de intervenções para determinar seus efeitos sobre desfechos específicos nas mulheres sobrevivente de câncer de mama, o que dificulta uma padronização e a generalização dos resultados.

Finalmente, cabe reafirmar a necessidade da conscientização de todos os profissionais de saúde envolvidos, incluindo médicos, profissionais de educação física, fisioterapeutas, psicólogos e nutricionistas sobre a importância do estímulo a estas mulheres à realização regular e continuada de exercícios físicos no pós-tratamento de câncer de mama, ressaltando-se os benefícios e ótimo custo-efetividade.

## Conclusão

A prática regular de exercício físico/atividade física deve ser estimulada, visando a prevenção primária do câncer de mama, melhoria da qualidade de vida e redução da mortalidade nas sobreviventes, mas os estudos não apresentam força de evidência no controle da doença. É importante ressaltar também o importante papel dos EF na redução da incidência das DCV, tornando-se fundamental o incentivo às mulheres se tornarem ativas. A observação de algumas especificidades na prescrição dos EF é necessária nas pacientes com câncer de mama, mas em linhas gerais, é semelhante à realizada para a população em geral. Estudos futuros são necessários para melhor nortear a prescrição individualizada destas pacientes.
