# Supplementary material for: Os Benefícios dos Exercícios Físicos no Câncer de Mama
Source: Arq Bras Cardiol. 2022 Nov 23;119(6):981–90. [Article in Portuguese] doi: 10.36660/abc.20220086 (PMC9814800; doi:10.36660/abc.20220086)
Supplement: Translation

# Translation: The Benefits of Exercise in Breast Cancer

**Authors:** Milena dos Santos Barros Campos, Roberta Helena Fernandes Feitosa, Carolina Christianini Mizzaci, Maria do Rosário Toscano von Flach, Betty Janny Maia Siqueira, Luiz EduardoMastrocola

**Affiliations:** 1 Aracaju SE Brazil Clínica e Hospital São Lucas, RedeD'Or São Luiz, Aracaju, SE – Brazil; 2 Aracaju SE Brazil Divisão de Cardiologia do Hospital Universitário de Sergipe, Aracaju, SE – Brazil; 3 Goiânia GO Brazil Gerência dos Sistemas de Informações Epidemiológicas – Secretaria Municipal de Saúde, Goiânia, GO – Brazil; 4 São Paulo SP Brazil Instituto Dante Pazzanese de Cardiologia, São Paulo, SP – Brazil; 5 Salvador BA Brazil Fundação Bahiana para Desenvolvimentodas Ciências (FBDC), Salvador, BA – Brazil; 6 Recife PE Brazil Universidade de Pernambuco, Recife, PE – Brazil

**Keywords:** Breast Neoplasms, Exercise Movement Techniques, Exercise, Diet, Healthy

## Abstract

Breast cancer is the most prevalent cancer among women, accounting for nearly 30% of all cancers, while in men, it represents only 1% of cases. Breast cancer is the main cause of death for cancer, and its incidence and mortality vary according to patients' ethnicity, geographic region, and socioeconomic status. Due to the low prevalence of breast cancer among men and the scarcity of studies in the literature, exercises have been prescribed based on extrapolations from studies on female patients. Scientific evidence has suggested beneficial effects of physical exercises on breast cancer prevention, treatment, and post-treatment. In addition to combatting sedentary behavior, it is essential to maintain a healthy body weight, limit alcohol consumption, and follow a balanced diet, rich in fruit, vegetables, grains and fibers, and limited in red meat. The effects of exercises are not restricted to breast cancer, but extend to controlling modifiable risk factors, and reducing the incidence of cardiovascular diseases, and all-cause and cardiovascular mortality.

## Introduction

Breast cancer is the most incident cancer among women in the world, with approximately 2.3 million new cases in 2020, corresponding to 24.5% of all types of cancer.^1^ In Brazil, apart from non-melanoma skin cancer, breast cancer is the most common among women, with the highest rates in the south and in the southeast regions.^2^ The highest occurrence in these regions may be attributed to the highest human development index and life expectancy, high prevalence of white race, life style, later pregnancy and less children.^3,4^ For 2022, it is estimated 66,280 new cases of breast cancer in Brazil.^2^

With respect to mortality, breast cancer is the main cause of death for cancer among the female population in Brazil, except for the north region, where colon cancer ranks the first. The highest mortality rates are observed in the south and southeast regions and the rates increase from the age of 40 years of age.^5^ In places where mortality rates are low, like the north of Brazil, the possibility of underdiagnosis of breast cancer cannot be excluded.

Genetic causes, including BRCA1 and BRCA2 mutations, are responsible for 5-10% of all cases of breast and ovarian cancer, with a greater contribution of environmental factors and lifestyle in the pathogenesis of these tumors.^6^ The BRCA 1 and BRCA2 genes produce tumor suppressive proteins. These proteins repair damaged DNA and thereby play a role in maintaining genetic material stability in each cell. When mutation or alteration occurs in one of these genes, the activity of the protein product can be altered, and the DNA damage may be not properly repaired. As a result, the cells are more likely to develop genetic changes that may lead to cancer development.^7^

The adoption of a healthy lifestyle is important for prevention of breast cancer, including an adequate diet (higher consumption of fruit, vegetables and whole grains, and lower consumption of red meat), weight control, reduction of alcohol intake, and regular physical exercises (PE).^8^ The effects are not restricted to the prevention, but also to disease control, since experimental studies have demonstrated influence on tumor formation kinetics, growth and recurrence.^9^

The third consensus of the World Cancer Research Fund^10^ and the Brazilian Society of Oncology guidelines on physical activity^11^ address the importance of women being physically active, involving several types of physical activities, from household chores (e.g. gardening), occupational, and recreational activities, to those systematically categorized as PE, whose frequency, intensity, time and type (aerobic, resistance and combined) are determined by prescription.

### Mechanisms of action of physical exercises in tumor progression

PE promote different organic and biological mechanisms that can be involved in the control of the development of several tumors. These responses originate from metabolic and hormonal changes, in addition to modulation of systemic inflammation.^12^ However, the potential of directly affecting tumor progression has been recently related to changes in vascularization and blood flow in tumors,^13^ the use of substrates by neoplastic cells, protein interactions between cancer and muscle tissue, and to the regulation of immune function by PE.^14–16^

Tumor microenvironment acts in the cooption and deviation of the action of immunoinflammatory and stromal cells.^17^ While the acute and transient action of lymphocytes and macrophages is a controlling and repair factor of tissue damage, chronic inflammation and macrophage infiltration into the tissue promote tumor progression.^18^

The development of anticancer therapy has been based on hallmarks (biological capabilities acquired by human cells during the development of tumors) proposed by Weinberg in 2000.^19^ Also based on these concepts, possible mechanisms of action and adaptation by which PE can influence these marks of tumor development have been studied (Figure 1).

**Figure 1:** Scheme of the mechanisms by which physical education can affect the hallmarks of cancer in breast cancer. Adapted from “Hallmarks of cancer”, by BioRender.com

Results of preclinical studies indicate that these molecular effects resulting from each exercise session overcome the control of hormonal factors and insulin.^20^ During exercise, these factors act immediately on tumor metabolism, and long-term training leads to metabolic and immunogenic adaptations that contribute to slow tumor progression.^21^

Evidence that PE inhibits malignant tumor progression emerged from animal models.^12,22,23^ Recently, clinical studies have identified cellular and molecular actions that are similar to exercise in patients with cancer, including breast cancer.^24,25^ However, there are still no studies that clearly determine the clinical relevance of these findings.

### Physical exercise in breast cancer prevention

One of the first and largest prospective studies on PE and breast cancer was the “Nurses Health Study”. This study evaluated 121 701 nurses aged between 30 and 55 years, during a follow-up period of 16 years, and showed that women who engaged in moderate or vigorous physical activity for seven or more hours per week had a nearly 20% lower risk of breast cancer than those who engaged in such physical activity for less than one hour per week (relative risk 0.82; 95% confidence interval [CI] 0.70-0.97). This benefit was observed in both premenopausal and postmenopausal women.^26^

Literature has suggested that women who exercise regularly have 10-25% lower risk of breast cancer compared with those who do not.^27,28^ This association seems to be stronger for activity sustained over the lifetime and after menopause, for women who are normal weight, have no family history of breast cancer, and are parous.^29^ Available evidences of the impact of PE on breast cancer reduction in BRCA1 and BRCA2 mutation carriers are limited, and larger studies are warranted.^30^

A meta-analysis published in 2013 of 31 prospective studies found a significant association between physical activity and reduction of breast cancer risk, with a combined relative risk (RR) with 95 % CI of 0.88 (0.85-0.91), highlighting the importance of prevention. Dose-response analysis suggested that the risk of breast cancer decreased by 2 % for every 25 metabolic equivalent (MET)-h/week increment in non-occupational physical activity (approximately 10 hours per week in household activities), 3 % for every 10 MET-h/week increment in recreational activity (equivalent to 4 h/week of walking in 3Km/h), and 5 % for every 2 h/week increment in moderate and vigorous recreational activity.^31^ Other studies have corroborated these results, suggesting greater risk reduction of breast cancer with higher levels of PE.^28,32^ The exact dose and the type of exercise needed to reduce breast cancer risk have not been clearly determined.

In an umbrella review^33^ about physical activity and cancer incidence and mortality, the results suggested a reduction of breast cancer risk in the general population. However, classification of physical activity across studies were heterogeneous and most reviews were based on observational studies, mainly cohort studies, in which the control of selection bias is difficult, since healthy habits tend to cluster. For example, a person with a healthy lifestyle eat well, has close to ideal body weight and is nonsmoker. To minimize limitations of this type of study, guidelines have been established based on cohort studies with larger samples of the population.^33^

The role of PE in breast cancer prevention seems to be linked to reductions in estrogen activity, insulin resistance, inflammation, and oxidative stress.^34^ Estrogen is related to reduction of cellular proliferation and tumor development. PE increase sex hormone-binding globulin, and reduce circulating levels of estrogen, as described in Figure 1. They contribute to the reduction of fat mass, mainly by visceral fat reduction, improvement of cellular insulin sensitivity and consequent decrease of insulin serum levels. Insulin is involved in the activation of aromatase and estrogen elevation, in addition to exerting mitogenic effects. In addition, PE have immunomodulatory effects, increasing both innate and acquired immunity and improving DNA repair mechanisms, thereby reducing the risk of breast cancer.^35^

More research is needed to fully understand the mechanisms by which physical activity can reduce breast cancer risk.

### General recommendations for physical activity in the prevention and control of breast cancer

In 2020, the World Health Organization (WHO) and the Brazilian Ministry of Health recommended for the general adult population (18-64 years old) and for breast cancer survivors, 150-300 minutes of moderate-intensity physical activity, at least 75–150 minutes of vigorous-intensity aerobic physical activity; or an equivalent combination of moderate- and vigorous-intensity activity throughout the week.^11,36,37^

Table 1 summarizes the description of different intensities of physical activity based on the Physical Activity Guidelines for the Brazilian Population.

**Table 1:** Description of physical activity intensities for the prevention and control of breast cancer

Intensity	Description	Examples
Light <3 metabolic equivalents (METs)	Requires minimum effort, with small increase in HR and RR increase. On a 0-10 scale, perceived exertion ranges from 1 to 4. One can breathe calmly, talk, or even sing during exercise	Standing or sitting, washing dishes, doing arts and crafts
Moderate 3 – 5.9 METs	Requires greater physical effort, with perceptible but moderate increments in RR and HR. On a 0-10 scale, perceived exertion ranges from 5 to 6. One can talk with difficulty but not sing	Walking at > 5 Km/h; cycling at < 5 Km/h, double tennis, and ballroom dancing
Vigorous > 6 METs	Requires great physical effort with large increments in RR and HR. On a 0-10 scale, perceived exertion ranges from 7 to 8. One is not able to talk duringthe exercise.	Running, slope walking, bicycling at > 16 km/h, aerobic dancing

Source: Adapted from Physical Activity Guidelines for the Brazilian Population.37 HR: heart rate; RR: respiratory rate.

It is important to differ physical activities (voluntary body movements, with energy expenditure resting levels) from PE (planned, structured, and repetitive physical activity, essentially aimed to improve cardiorespiratory fitness, strength, flexibility and balance). It is recommended that PE be supervised by a physical educator or a physical therapist, and all programs include aerobic components (walking, cycling, dancing, jogging, swimming), muscle-strengthening activities (strength training, Pilates, functional exercise) and range of motion exercises (stretching, yoga, tai-chi).^11^

Aerobic exercises increase the levels of peripheral beta-endorphins, which are correlated with decreased systemic sympathetic activity and improvement in serotonergic activity, reflected in the activity of neuromuscular junctions. Resistance training exercise promote better synchronization, recruitment and excitability of motor units. Finally, flexibility exercises can lead to a better control of articular structures and soft parts.^38,39^

Now we describe particularities of physical activities and PE during treatment and follow-up periods of survivors after breast cancer treatment.

### Physical exercises during breast cancer treatment

Breast cancer treatment should be individualized according to patients’ age, hormonal status, comorbidities, lifestyle, and personal choices, as well as to fundamental pillars in determining the prognosis: the extent of the disease (cancer staging) and the type of the tumor. In general, treatment can be divided into local therapy (surgery, radiotherapy, and breast reconstruction) and systemic therapy (chemotherapy, hormone therapy and biological therapy).^40^

Chemotherapy is associated with fatigue, anorexia, anemia, neutropenia, thrombocytopenia, peripheral neuropathies and, in some cases, cardiotoxicity.^41,42^ The side effects of hormone therapy include weight gain, arthralgia, myalgia, bone loss, effects on the cardiovascular system and changes in the lipid profile.^43^ Sequelae of radiation therapy include cardiac and pulmonary damage, lymphedema, brachial plexopathy and secondary malignant diseases.^44^ Associated to these physical repercussions, emotional changes including depression, anxiety, low self-esteem, and negative body image can occur, since the disease affects an important symbol of femininity, sexuality and maternity.^45^

Six months after the diagnosis, approximately 90% of women present at least one of the adverse symptoms of the antineoplastic therapy; 60% have multiple symptoms that affect not only patient therapy and quality of life, but also survival rates. Six years after treatment, up to 30% of women still have complaints related to the therapies.^46^

Physical activity is safe and can be performed at different stages of cancer treatment, resulting in better quality of life, and improved global function, and lower psychological symptoms related to disease and its treatment.^47,48^

Pain is one of the most common symptoms in breast cancer patients; 30-60% of patients have moderate to severe symptoms, that may lead to activity restrictions and limitations in physical activity during and after therapeutical interventions.^39,49^ Pain manifestations tend to decrease with physical training, with direct implications for strength gain, better cardiorespiratory capacity and flexibility, and also for lower rates of fatigue, length of hospital stay, anxiety, depression, sleep disorders, nausea and vomiting.^50,51^ Figure 2 summarizes the final clinical effects of PE during treatment and in other breast cancer stages.

**Figure 2:** Clinical results of exercise in breast cancer timeline.

Van Waart et al.^52^ demonstrated that, in patients undergoing chemotherapy, an aerobic training program was associated with enhanced physical functioning, with maintenance of cardiorespiratory fitness, facilitating the return to work during and after treatment, in addition to reducing the incidence of nausea and vomiting as with the group that did not undergo physical training.^52^

In addition, a multicentric study^53^ evaluated the effect of physical activity in 301 patients during breast cancer chemotherapy and demonstrated improvements in health-related fitness in the three groups of exercise intervention: STAN group (three sessions of 25–30 min/week of vigorous-intensity aerobic exercise), HIGH group (three sessions of 150 min/week of vigorous-intensity aerobic exercise, and the COMB group (aerobic exercise plus a resistance exercise program). The higher dose of aerobic exercise intervention (HIGH group) was more effective in improving life quality and aerobic capacity, and in controlling pain and endocrine symptoms (e.g. hot flashes). However, the COMB group was superior to the HIGH and STAN groups in muscular strength gain.^53^

In chemotherapy patient, resistance exercise programs are associated with improved self-esteem, muscular strength, and body composition, without causing or aggravating lymphedema or other complications in those who underwent surgery.^54,55^ Before initiating upper limbs exercises, it is recommended to assess the mobility of the arms. In addition, specific examination for the presence of peripheral neuropathies, musculoskeletal disorders, and risk of fractures, especially in patients on hormone therapy and in those with metastatic bone disease is advisable.^46,56,57^

The combination of the three exercise programs proposed (aerobic, resistance and flexibility exercise) have largely contributed to the control of pain and fatigue. Improvement of cardiorespiratory function, due to increased aerobic capacity (maximal oxygen consumption) in combined exercise training programs may be explained by the ventilation perfusion matching and oxidative capacity of skeletal muscle.^55^ This can play an important role in the management of structural disorder related to chemo- and radiation-induced toxicity.^46^

Chemotherapeutic agents that also cause direct and indirect cardiotoxicity, with acceleration of general and vascular aging, and consequent decline of cardiopulmonary reserve. Both the disease and the therapy can contribute to weight gain and reduction of physical activity,^58^ potentially increasing the risk for cardiovascular diseases (CVD). Studies on secondary prevention have corroborated the improvement of cardiopulmonary function with physical training programs in breast cancer women.^59^

Cardiotoxicity, associated with psychoemotional factors, affects autonomic balance and consequently cardiovascular mortality.^60^ In patients treated at initial stages of the disease, a sustained increment in sympathetic function and reduction in parasympathetic effect on the sinoatrial node have been reported.^61^ Other reports have pointed out a reduction in heart rate variability and baroreflex sensitivity among women with a history of breast cancer.^61–63^

In addition to autonomic regulation, another important factor for the development of CVD is endothelial dysfunction. A recent meta-analysis^64^ evaluated 163 patients from four studies (two on breast cancer, two on prostate cancer). Aerobic exercise improved vascular function and peak oxygen consumption. These data reinforce the importance of PE as an adjuvant therapy in breast cancer treatment, especially regarding the management of the side effects.

With respect to overall and specific mortality, evidence accumulated so far suggests a favorable effect of moderate to vigorous physical activity, and preliminary evidence indicates associations of physical activity with risk reduction of breast cancer recurrence and progression.^11^ A recent study evaluated systematic reviews on physical activity and reduction of all-cause mortality and breast cancer mortality, considering the dose-response relationship (also regardless of body mass index); the certainty of evidence was moderate. Regarding associations of the domain or the type of physical activity with mortality, the certainty of evidence was classified as low and, so far, it was not possible to identify physical activity modalities that had the greatest impact on the outcome. The same study evaluated risks and benefits, patients’ values and preferences, resources required to meet the recommended physical activity, equity, acceptability of the recommendation, and the strength of the recommendation of PE to increase breast cancer survival was classified as “strong”.^11^ Future studies may change the quality of available evidence; it remains an open field of research.

### Physical exercises in breast cancer post-treatment

PE have been strongly recommended for breast cancer survivors and associated not only with improvement of quality of life, but also with possible increased survival.^65–67^ A prospective study that included 2987 women with stage I, II, or III breast cancer between 1984 and 1988, followed up until death or June 2002, showed that physical activity after breast cancer diagnosis can reduce the risk of death for the disease. The greatest benefit occurred in women who performed the equivalent of walking three to five hours per week at an average pace.^65^

After the end of treatment, the main objective is to rehabilitate patients to return to their usual activities. Regular exercises can contribute to physical and psychological well-being and improved quality of life, consisting of one of the main recommendations to prevent chronic degenerative conditions; it would not be different for patients who had recently faced cancer treatment.^68^ Evidence has shown that PE have a positive impact on survival and minimize breast cancer-related morbidity.^65^ Despite favorable data, the practice of PE is limited by barriers like fatigue, lack of motivation, loss of self-confidence, inadequate follow-up, lack of family support and lack of instructions.

Encouraging women in the post-treatment period to adopt a healthy lifestyle – by avoiding excess alcohol and increasing fruit and vegetable intake and physical activity volume, is important to improve their quality of life and health.^69^ Increased exercise levels represent a modifiable health behavior that can ameliorate sequalae of the disease and help women to return to their health status before cancer diagnosis and treatment.^70^ Thus, current recommendations of PE for breast cancer survivors are based on the return, as soon as possible, to habitual daily activities, on the maintenance of metabolic expenditure during and after therapies, and on the classical recommendation of weekly aerobic exercises.^57^

Also, it has been demonstrated that physical inactivity is related to weight gain after the diagnosis which, in turn, has been associated with lower survival in some studies.^71,72^ More physically active women are less likely to gain weight after the diagnosis, improving the chance of survival.^65,73^

Obesity is related to increased mortality rates for breast cancer (13-20%) and all-cause mortality (14-70%).^74–77^ Obesity was also associated with a twice greater chance of post-menopausal contralateral breast cancer and a nearly 60% greater occurrence of other cancers.^76^ Therefore, maintaining normal body mass index can reduce the risk of a new breast cancer in the postmenopausal period, other cancers, and all-cause mortality.^75,76,78^

Giallauria et al.^61^ evaluated whether exercise training improved autonomic function in women with a history of primary invasive breast cancer. Fifty-one patients enrolled in the DIANA clinical trial were divided into two groups. Group 1 (n = 25) that followed a formal exercise program of moderate intensity – three sessions/week on a bicycle or treadmill at 70 ± 2% VO_2_ peak for 12 weeks, followed by one session/week until one-year follow-up. Group 2 while a control group (n = 26) did not perform any formal training. At baseline and at one-year follow-up, all patients underwent cardiopulmonary exercise stress test. Heart rate recovery (HRR) was calculated as the difference between heart rate at peak exercise and heart rate at the first minute of recovery. Compared with control group, group 1 showed significant improvement of VO_2_ peak (from 12.4 ± 2.9 to 14.3 ± 3.3 mL/kg/min; p<0.001) and in HRR (from 17.6 ± 6.4 to 23.0 ± 8.3 beats/min; p<0.001). The authors concluded that moderate-intensity exercise is associated with improvement of autonomic function in breast cancer survivors.^62^

Evidence suggests that PE can also promote physiological and psychological benefits in cancer survivors.^70,79^ A meta-analysis of randomized controlled trials by Fong et al.^70^ reported that PE had positive effects on physical functions, body weight and quality of life in patients after treatment for breast cancer.^70^ Additionally, results from another systematic review^66^ indicated that PE can have beneficial effects on overall and on certain domains of quality of life, like body image, self-esteem, emotional well-being, sexuality, sleep disorder, social functioning, anxiety, fatigue and pain.^66^ Also, a Cochrane database systematic review that included 63 trials and 5761 women evaluated the effects of PE in patients in the post-treatment period and in a control group. Once again, it was shown that that physical activity interventions resulted in improvements in quality of life, emotional health, anxiety, physical function, muscular strength, and fatigue. Besides, relatively few adverse events were reported in the trials, suggesting that PE are safe in this population.^67^

To be safe, exercise prescription requires the understanding, by the multidisciplinary team (physical educators, physical therapists, among others), of the peculiarities, implications and consequences of cancer treatment.^57^ Prescriptions should be made according to pre-treatment physical performance and comorbidities of cancer survivors, therapeutic response and negative immediate and persistent effects of treatment.^57^ Special attention should be given to peripheral neuropathies and secondary musculoskeletal diseases, regardless of the treatment time. Patients on hormone therapy should be assessed for the risk of fractures. It is also recommended evaluating the mobility of arms and shoulders before initiating upper limb exercises. It is important to consider the needed time for wound healing, which may be eight weeks or longer in mastectomies.^57^

Individuals with bone metastatic disease will required individualized exercise programming aiming at determining safety limits before initiating the PE. Rehabilitation of these patients include adaptations in pre-established programs, with reductions in impact, intensity, and volume, due to the increased risk posed by bone frailty and fractures.^57^ In addition, individuals with known CVD (secondary to cancer or not) also require initial individualized examination regarding the safety of the exercise programs, closer supervision, and shorter intervals. Guidelines’ recommendations on exercise and rehabilitation should be followed, especially considering cardiovascular and pulmonary contraindications.^57^

Cancer survivors should be physically active. However, exercise prescription including exercise frequency, intensity, type and duration has been based on limited literature data. Table 2 summarized recommended exercise prescription for breast cancer patients in the post-treatment.^47,57^

**Table 2:** Recommended prescription of physical exercises in the post-treatment of breast cancer

	Frequency	Intensity	Duration/performance	Quality
Aerobic	Patient should start exercises twice a week and progressively increase until 3-5 times a week	Patient should be instructed about effort perception. If exercises are well tolerated (without symptoms or side effects), the intensity of exercises should not be different from that in healthy population. Intensity should be from moderate to vigorous	Exercise duration should be increased according to patient tolerance. A target of 75min/week of vigorous exercise or 150min/week of moderate exercise should be aimed	Rhythmic, prolonged exercises that use large muscle groups. Examples: swimming, walking, bicycling, dancing
Resistance	2-3 days a week	Moderate intensity (60-70% maximum repetitions)	Sets of 8 - 12 repetitions	Programs should include weights, resistance training equipment, functional tasks with weights, using the main large muscle groups
Flexibility	Exercises can be performed daily, according to patient's condition			Programs should include stretching exercises. Attention to body parts with restricted mobility due to treatment

Source: Adapted from the American College of Sports Medicine guidelines for exercise testing and prescription.^*47*^

The progression of PE should be slower in cancer survivors as compared with healthy individuals, particularly if the prescribed exercises result in greater fatigue and unexpected adverse effects, which serves as an alert to individual's capacity thresholds. There are no maximum loads for weight training exercises for these patients. Attention should be paid to symptoms in the arms and shoulders, including lymphedema, resulting in load decrease or interruption of specific exercises according to the symptom reported.^57^

Despite all the benefits of regular physical activity described above, there is no consensus or clear standard of the magnitude of their benefits, the way of administration or the most effective PE for this population. Further research is needed to establish the ideal exercise prescription. Studies so far have evaluated the effects of different exercise modalities, frequencies, intensities and durations on specific outcomes in breast cancer survivors, which make generalization and standardization of results difficult.

Finally, we reinforce the need of all health professionals involved – physicians, physical educators, physical therapists, psychologists, and nutritionists – be aware of the importance of encouraging these women to regularly exercise after breast cancer treatment, highlighting the benefits and excellent cost-effectiveness.

## Conclusion

Regular exercise/physical activity should be encouraged among women, targeting primary prevention, improvement of life quality and reduction of mortality among survivors, although studies have not reported the strength of evidence for breast cancer control. It is also important to highlight the important role of PE in reducing the incidence of CVD, which reinforces the importance of encouraging these women to be physically active. Although attention should be paid to some details in exercise prescription to breast cancer patients, in general, it is not very different from that made to the general population. Future studies are needed to better guide individualized prescriptions for these patients.
